# TGR5 expression in normal kidney and renal neoplasms

**DOI:** 10.1186/s13000-018-0700-5

**Published:** 2018-04-02

**Authors:** Chaohui Lisa Zhao, Ali Amin, Yiang Hui, Dongfang Yang, Weibiao Cao

**Affiliations:** 1 Department of Pathology, Rhode Island Hospital and the Warren Alpert Medical School of Brown University, 593 Eddy Street, APC 12, Providence, RI 02903 USA; 2 Department of Medicine, Rhode Island Hospital and the Warren Alpert Medical School of Brown University, Providence, RI 02903 USA

**Keywords:** G protein-coupled bile acid receptor, TGR5, Renal cell carcinoma, Urothelial cell carcinoma, Oncocytoma

## Abstract

**Background:**

The G protein-coupled bile acid receptor (TGR5) is a cell surface receptor which induces the production of intracellular cAMP and promotes epithelial-mesenchymal transition in gastric cancer cell lines. TGR5 is found in a wide variety of tissues including the kidney. However, the patterns of TGR5 expression have not been well characterized in physiologic kidney or renal neoplasms. We explore the expression of TGR5 in benign renal tissue and renal neoplasms and assess its utility as a diagnostic marker.

**Methods:**

Sixty-one renal cortical neoplasms from 2000 to 2014 were retrieved. TGR5 protein expression was examined by immunohistochemistry. TGR5 mRNA was also measured by real-time PCR.

**Results:**

In normal renal tissue, TGR5 was strongly positive in collecting ducts, distal convoluted tubules and thin loop of Henle. Proximal convoluted tubules showed absent or focal weak staining. In clear cell renal cell carcinomas (RCCs), 25 of 27 cases (92%) were negative for TGR5 (*p* < 0.001). TGR5 mRNA was also significantly decreased in clear cell RCCs, suggesting that decreased TGR5 protein expression may be attributable to the downregulation of TGR5 mRNA in these tumors. All 11 papillary RCCs expressed TGR5 with 45% (5/11) exhibiting moderate to strong staining. All chromophobe RCCs and oncocytomas were positive for TGR5 with weak to moderate staining. TGR5 mRNA expression in these tumors was similar to normal kidney. All urothelial carcinomas of the renal pelvis strongly expressed TGR5 including a poorly differentiated urothelial carcinoma with sarcomatoid features.

**Conclusion:**

TGR5 is strongly expressed in collecting ducts, distal convoluted tubules and thin loop of Henle. TGR5 protein and mRNA expression were notably decreased in clear cell RCCs and may be helpful in differentiating these tumors from other RCCs.

## Background

Renal malignancies are the 7th most common cancer in men in the US with approximately 14,000 attributable deaths annually [1]. Malignant renal cell carcinomas (RCCs) include clear cell RCCs, papillary RCCs (type 1 and type 2), chromophobe RCCs, collecting duct carcinomas, clear cell papillary RCCs, and others [2]. Benign renal epithelial neoplasms include papillary adenomas, oncocytomas and metanephric adenomas [2]. Overall, RCCs constitute 80–85% of primary renal neoplasms. Urothelial cell carcinoma of the renal pelvis is the second most common malignant neoplasm in the kidney [1]. Morphologic similarities among these tumors may present difficulties in accurately classifying these lesions which follow different clinical outcomes.

The G protein-coupled bile acid receptor (TGR5) is a cell-surface receptor mediating bile acid effects [3]. Both primary and secondary bile acids bind to TGR5 to induce cyclic AMP (cAMP) production [3]. In turn, TGR5 has been shown to activate a MAP kinase signaling pathway [4] and to be linked to an increase in the intracellular ATP/ADP ratio [5]. It plays an important role in energy homeostasis, bile acid-regulated lipid metabolism, and glucose metabolism [6, 7] . TGR5 is also implicated in the mitochondrial response to inflammation and the development of fibrosis in the kidney [8, 9]. TGR5 expression is present in a variety of tissues including kidney [9–11]. Some studies have reported strong TGR5 expression in renal tubular cells [10, 11] as well as the mesangial cells of rat kidney [10]. However, its distribution of expression has not been well characterized. Renal tumors have also not been interrogated for TGR5. In this study, we showed that the TGR5 receptor was strongly expressed in distal convoluted tubules, thin loop of Henle and collecting ducts and that TGR5 lost expression in clear cell RCCs.

## Methods

### Case selection

With Institutional Review Board approval at Rhode Island Hospital, sixty-one renal cortical neoplasms were identified from the archives of the Department of Pathology between 2000 and 2014. These included 27 clear cell RCCs and 11 papillary RCCs, 8 of which were type 1 and 3 of which were type 2. The remainder of the cases consisted of 8 papillary urothelial carcinomas including one poorly differentiated case involving the cortex, 6 chromophobe RCCs, 5 oncocytomas, 2 clear cell papillary RCCs, 1 metanephric adenoma, and 1 poorly differentiated RCC with sarcomatoid features. Formalin-fixed paraffin-embedded tissue sections and blocks were retrieved. The corresponding hematoxylin-eosin slides were reviewed for confirmation of diagnosis and adequacy of material by a specialist in genitourinary pathology (AA). Multiple representative sections of each case were examined. Corresponding normal kidney control sections separate from the tumors were also reviewed for all cases. Snap-frozen tissue from normal kidney, clear cell RCCs, papillary RCCs, chromophobe RCC and oncocytomas was obtained from the Rhode Island Hospital tissue bank.

### Tissue microarray construction

Paraffin blocks containing areas of carcinomas were identified on the hematoxylin and eosin-stained sections. Areas of the tumor were identified and marked on the source block. The source block was cored using a 1-mm needle and the tissue was transferred to a recipient “master block” using a Beecher Tissue Microarrayer (Beecher Instruments, Silver Spring, MD). Three to six cores of each tumor were arrayed per specimen. Additionally, a core of normal kidney tissue was also sampled when present [12].

### Immunohistochemistry

Immunohistochemistry for TGR5 was performed on 4-μm paraffin sections of the tissue microarray or whole tissue sections. Slides were stained with TGR5 antibody (rabbit anti-human, polyclonal, 1:1000; Sigma-Aldrich, St. Louis, MO) using the DAKO Envision + Dual Link System and the DAKO Liquid 3,3′-Diaminobenzidine Substrate Chromogen System (DAKO North America, Carpinteria, CA). Bile ducts from liver tissue were used as positive controls. Negative controls were included by replacement of the primary antibody with non-reacting antibodies derived from the same species. The specificity of TGR5 antibody has previously been confirmed by Western blot analysis in our lab [12].

Tissue microarray was employed to study normal kidney and 44 cases of renal neoplasms, including all clear cell RCCs, 1 metanephric adenoma, 8 papillary urothelial carcinomas, 4 type 1 papillary RCCs, 3 oncocytomas, and 1 chromophobe RCC.

Immunohistochemistry was also performed on whole tissue sections for 17 cases including 4 papillary RCC (4 type 1 papillary RCC and 3 type 2 papillary RCC), 5 chromophobe RCC, 2 oncocytomas, 2 clear cell papillary RCC, and 1 poorly differentiated RCC with some sarcomatoid features.

### Immunohistochemistry assessment

Immunohistochemical results were evaluated semi-quantitatively within neoplastic tissue. Cells displaying strong intensity staining for TGR5 were scored as 3+, moderate staining as 2+, and weak staining as 1+. The extent of staining was scored as follows: 0 (< 5%), 1+ (5–25%), 2+ (26–50%), 3+ (51–100%). A combined score of intensity and extent was calculated and assigned as follows: weak staining 1–2, moderate staining 3–4, and strong staining 5–6. At least three cores were scored per case. Analysis of three cores per case in this fashion has been shown to be comparable to whole tissue sections [13]. All sections were independently scored by two pathologists (WC and CZ) blinded to clinicopathological features and outcome.

### Quantitative real time PCR

TGR5 mRNA was measured using real-time PCR analysis as we have previously described [14]. The primers used were as follows: TGR5 sense (5′- CTGGCCCTGGCAAGCCTCAT-3’), TGR5 antisense (5′- CTGCCATGTAGCGCTCCCCGT-3’), 18S sense (5**’**-CGGACAGGATTGACAGATTGATAGC-3**’**), and 18S antisense (5**’**-TGCCAGAGTCTCGTTCGTTATCG-3**’**). Reactions were carried out in an Applied Biosystems StepOnePlus real-time PCR system (Applied Biosystems, Foster City, CA) for one cycle at 94 °C for 5 min followed by 40 cycles at 94 °C for 30 s, 62 °C for 30 s, and 72 °C for 30 s, 1 cycle at 94 °C for 1 min, and 1 cycle at 55 °C for 30 s. Fluorescence values of SYBR Green I dye, representing the quantity of product amplified at that point in the reaction, were recorded in real time at both the annealing and extension steps of each cycle. The C_t_, defined as the point at which the fluorescence signal becomes statistically significant above background, was calculated for each amplicon for each sample using StepOne software (Applied Biosystems, Foster City, CA). The transcript level of each specific gene was normalized to 18S amplification.

### Statistical analysis

A Chi-square test was utilized to compare groups where appropriate. Data were expressed as mean ± SEM. Statistical differences between two groups were determined by Student’s t-test. Differences among multiple groups were tested using analysis of variance (ANOVA) and checked for significance using Fisher’s protected least significant difference test. A *p*-value of 0.05 or less was considered statistically significant.

## Results

### TGR5 protein expression

**Fig. 1:**
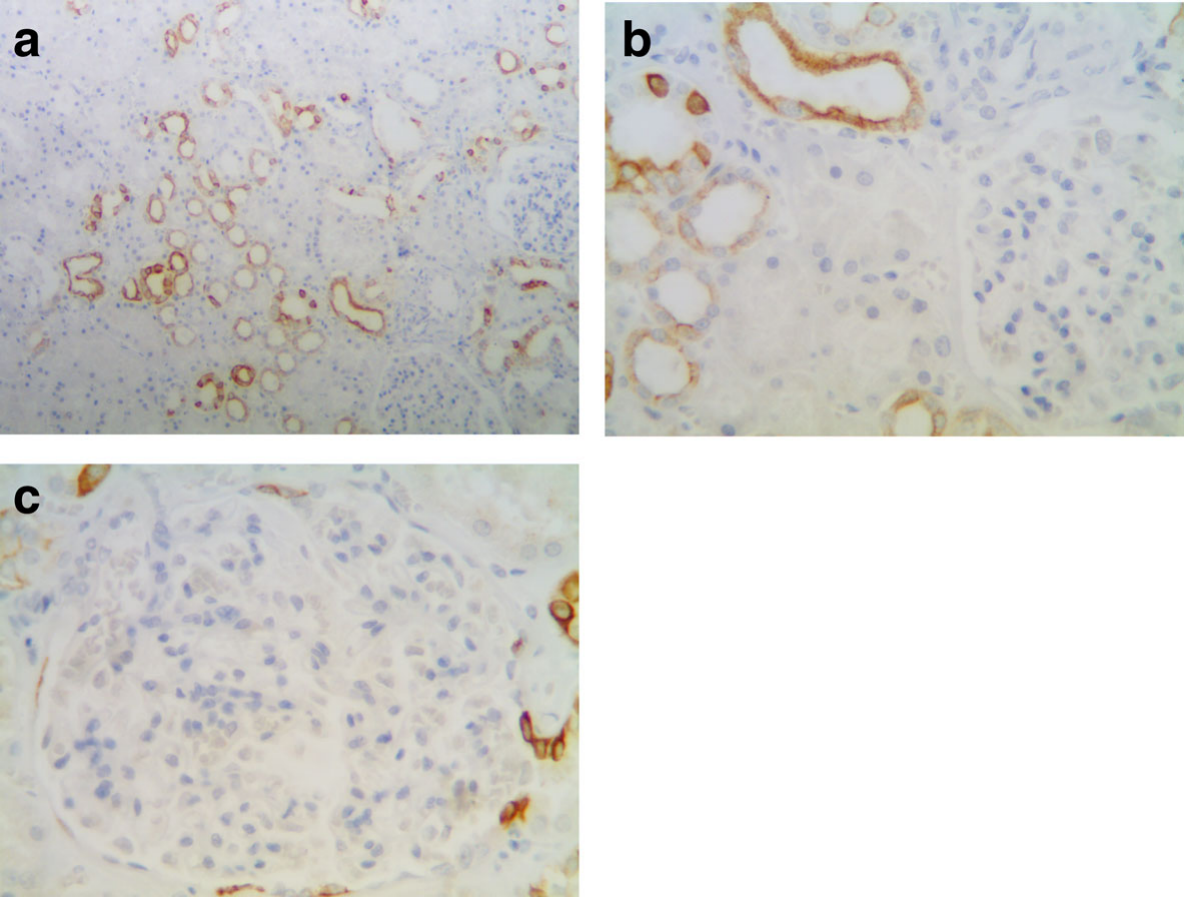
TGR5 staining in normal renal cortex. **a** TGR5 is strongly expressed in the distal convoluted tubule, thin loop of Henle, and collecting duct (100X). **b** Absent or only minimal focal weak staining is observed in the proximal convoluted tubular cells (arrow, 400X). **c** In the glomeruli, TGR5 expression is not identified in the glomerular tufts while staining is present focally in the parietal epithelial cells (400X)

In normal kidney, the proximal tubular cells are more columnar and eosinophilic than cells of the distal tubules on H&E slides. The ascending limb has no brush border and the cells are more cuboidal than adjacent proximal tubular cells [15]. According to this criterion, TGR5 was strongly expressed in the distal convoluted tubules, thin loop of Henle, and collecting ducts (Fig. 1a). In contrast, the proximal convoluted tubular cells showed absent or focal weak staining (Fig. 1b). TGR5 expression was not observed in the glomerular tuft, but focal immunoreactivity was identified in parietal epithelial cells (Fig. 1c).

**Fig. 2:**
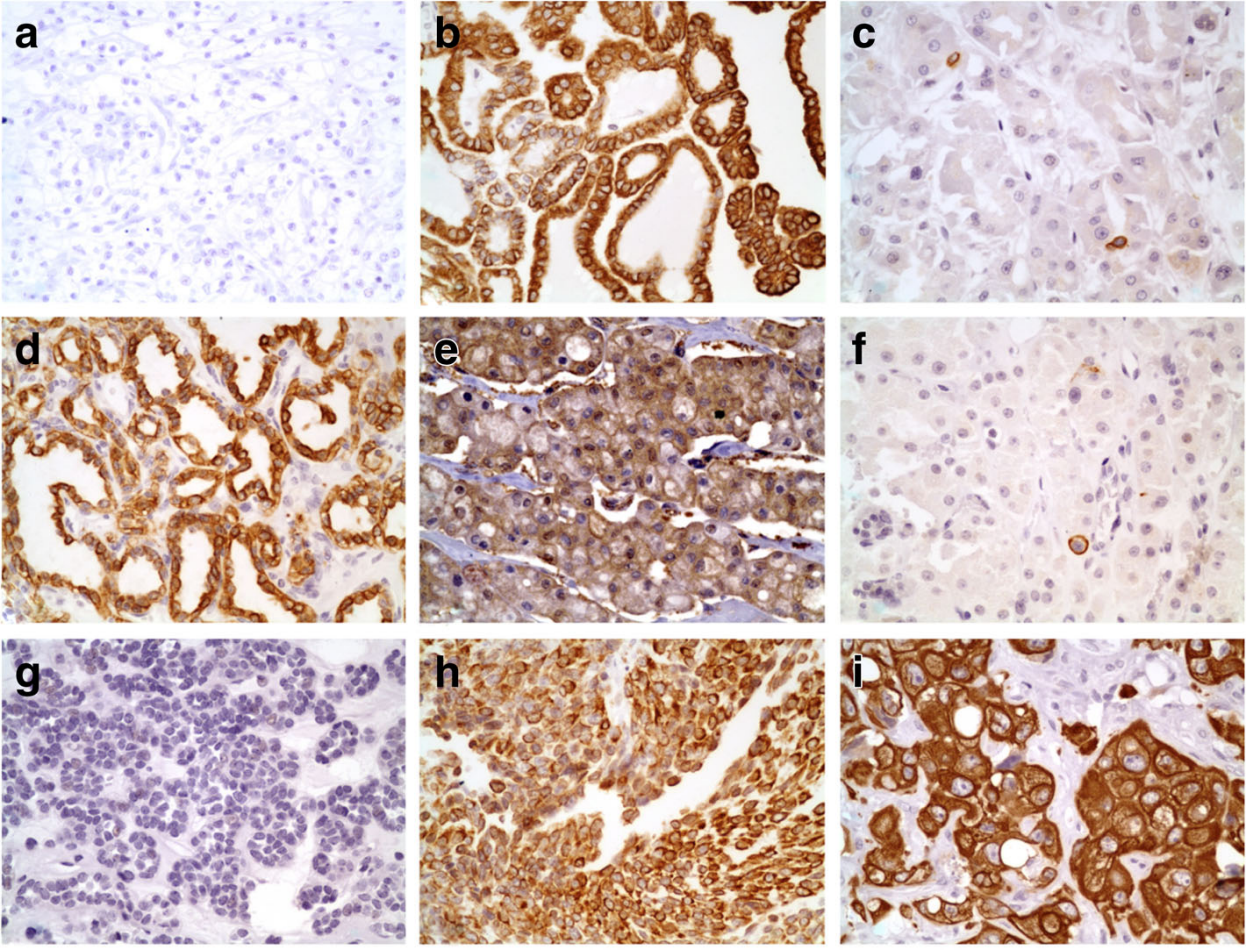
TGR5 staining - **a** Clear cell RCC: negative for TGR5 (400X). **b** Type 1 papillary RCC: strongly positive for TGR5 (400X). **c** Type 2 papillary RCC: weakly positive with focal cells expressing TGR5 (400X). **d** Clear cell papillary RCC: strongly positive for TGR5 (400X). **e** Chromophobe RCC: moderate expression of TGR5 (400X). **f** Oncocytoma: weakly positive with focal cells expressing TGR5 (400X). **g** Metanephric adenoma: negative for TGR5 (400X). **h** Urothelial carcinoma of the renal pelvis: strongly positive for TGR5 (400X). **i** Poorly differentiated urothelial carcinoma with sarcomatoid features: strongly positive for TGR5 (400X)

The expression of TGR5 in renal cell neoplasms is summarized in Table 1. Notably, among 25 cases of clear cell RCCs, 92% (25/27) of cases were negative for TGR5 staining (*p* < 0.001, Fig. 2a). The remaining 2 cases of clear cell RCCs (2/27) exhibited TGR5 focal staining in areas with papillary features. One of these two TGR5-positive cases also exhibited sarcomatoid differentiation. Both cases of clear cell papillary RCCs (2/2) displayed strong TGR5 staining (Fig. 2d).

**Table 1:** The expression of TGR5 in RCCs

	Case number (N)	Negative	Weak	Moderate	Strong
Clear cell RCC	27	25 (92%)	0	1 (4%)	1 (4%)
Papillary RCC	11	0	6 (55%)	4 (36%)	1 (9%)
Type 1	8	0	3 (37%)	4 (50%)	1 (13%)
Type 2	3	0	3 (100%)	0	0
Papillary urothelial carcinoma	8	0	1 (12%)	0	7 (88%)
Clear cell papillary RCCs	2	0	0	0	2 (100%)
Chromophobe RCC	6	0	2 (33%)	4 (67%)	0
Oncocytoma	5	0	5 (100%)	0	0

All papillary RCCs (11/11) demonstrated TGR5 staining. The TGR5 staining in Type 1 papillary RCCs (8 cases) were variable from weak to strong staining (Fig. 2b). All 3 cases of type 2 papillary RCCs showed only focal weak staining (Fig. 2c). There were no statistically significant differences between type 1 and type 2 papillary RCCs (*p* = 0.152).

All chromophobe RCCs (6/6) exhibited weak to moderate TGR5 staining (Fig. 2e). The oncocytomas all (5/5) showed weak TGR5 expression (Fig. 2f). No statistically significant differences between chromophobe RCCs and oncocytomas (*p* = 0.082) were identified. The metanephric adenoma (0/1; Fig. 2g) and poorly differentiated RCC with sarcomatoid features (0/1) were negative for TGR5 staining.

Urothelial carcinomas all uniformly (8/8) expressed TGR5 (Fig. 2h) including the case of poorly differentiated sarcomatoid case (Fig. 2i).

### TGR5 mRNA expression

**Fig. 3:**
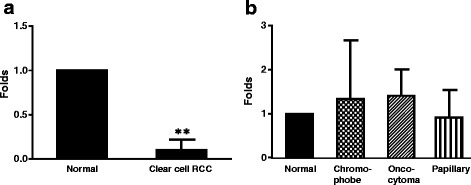
TGR5 mRNA expression. A. TGR5 mRNA was significantly decreased in clear cell RCC (*N* = 5, un-paired t test), when compared with normal renal tissue. The data suggest that decreased TGR5 protein expression may be due to downregulation of TGR5 mRNA in clear cell RCC. B. TGR5 mRNA expression in papillary RCCs, chromophobe RCCs and oncocytomas reveal no significant differences from normal renal tissue (*N* = 3–5, ANOVA)

TGR5 mRNA was significantly diminished in clear cell RCC (*N* = 5, *p* < 0.001, fig. 3a) in comparison to normal non-neoplastic renal tissue. TGR5 mRNA expression in papillary RCCs, chromophobe RCCs, and oncocytomas did not significantly differ from normal renal tissue (fig. 3b).

## Discussion

The distal convoluted tubules play a key role in regulating extracellular fluid volume and electrolyte homeostasis. Distal tubular cells are rich in mitochondria and biochemical studies reveal that distal tubular cells have a higher level of Na^+^/K^+^-ATPase activity than any other tubular segment [16–18]. Recently, TGR5 has been identified as a cell surface receptor, which can induce the production of intracellular cAMP and activation of a MAP kinase signaling pathway [4]. TGR5 has also been linked to an increase in the intracellular ATP/ADP ratio [5]. TGR5 agonist may be useful in the treatment of kidney injury and various metabolic diseases including diabetes and obesity [9]. Our data reveal that TGR5 is strongly positive in normal distal convoluted tubules, the thin loop of Henle, and collecting ducts. In comparing the glomeruli and proximal tubular cells, increased expression in the distal tubular cells corresponds to elevated Na^+^/K^+^-ATPase activity in the distal tubular cells. This might account for high TGR5 expression in the distal tubular cells in the kidney.

Immunohistochemical staining demonstrated no expression of TGR5 in the glomeruli with the exception of some parietal cells within Bowman’s capsule. This is consistent with prior studies in the literature [19]. Xiong et al. previously described TGR5 expression in rat mesangial cells [10]. This discrepancy may be due to one of three scenarios. First, there may be intrinsic differences in TGR5 expression between species. Second, the primary polyclonal antibody employed in the prior study was obtained from a different manufacturer which may have resulted in discrepant immunoreactivity. Third, TGR5 may be expressed in low levels in these cells which are undetectable by immunohistochemistry.

Strong expression of TGR5 in distal tubular cells and collecting ducts lends credence to the theory that some renal tumors such as papillary RCCs, chromophobe RCCs, and oncocytomas likely derive from distal convoluted tubules or collecting ducts as suggested by some studies [20, 21]. Electron microscopic and immunohistochemical data implicate the intercalated cells of the collecting duct as the cell of origin for chromophobe RCCs [22] and oncocytomas [23]. These tumors variably express TGR5. In contrast, clear cell RCCs are suggested to originate from proximal tubular cells [20] which are negative or focal very weakly positive for TGR5. TGR5 mRNA is significantly decreased in clear cell RCC corresponding to the diminished TGR5 protein expression, suggesting that decreased TGR5 protein expression may be attributable to the downregulation of TGR5 mRNA in these tumors.

Clear cell RCCs comprise 70% to 80% of all RCCs [24]. Clear cell papillary RCCs constitute a diagnostic challenge given their morphologic similarities to clear cell RCCs [25]. Carbonic anhydrase 9 (CA-IX) is expressed in both clear cell and clear cell papillary RCCs [25]. CA-IX is expressed in approximately 85% of clear cell RCCs [25]. Since the expression TGR5 is negative in up to 92% of clear cell RCCs, this marker may be useful in the diagnostic work-up of these tumors. Additional studies directly comparing TGR5 and CA-IX are warranted to better understand the utility of TGR5 in this context. Notably, TGR5 was strongly expressed in clear cell papillary RCCs although only 2 cases were included in our cohort.

Papillary RCCs are the second most common histologic subtype and comprise 7–15% of all RCCs [25]. Immunohistochemical markers such as CK7, alpha methyl acyl coenzyme A racemase (AMACR), and c-kit (CD117), have been leveraged to differentiate these tumors from clear cell RCCs. CK7 is expressed in 80–87% papillary RCCs and is variably positive in 0–37% of clear cell RCCs [24]. AMACR is positive in 80–100% of papillary RCCs and variably positive in 4–68% of clear cell RCCs [24]. C-kit (CD117) expression is variable in clear cell RCCs (0–5%), papillary RCCs (0–13%), chromophobe RCCs (82–100%), and oncocytomas (58–100%) [24]. We found that TGR5 is positive in all papillary RCCs including both type 1 and type 2. Oncocytomas and chromophobe RCCs also all expressed TGR5. However, only 8% of clear cell RCCs were positive. This supports the potential usefulness of the inclusion of TGR5 into the diagnostic work-up of these tumors.

Urothelial carcinomas of the renal pelvis including sarcomatoid type of poorly differentiated urothelial carcinomas strongly expressed TGR5. Although only one case poorly differentiated RCC with sarcomatoid features was included in our study, this case was negative for TGR5. This may suggest that TGR5 could be used to differentiate poorly differentiated urothelial carcinomas from poorly differentiated RCCs. Further studies with a larger cohort are necessary to establish the utility of TGR5 for this application.

Our cases suggest that TGR5 would have overall limited diagnostic utility in differentiating among non-clear cell RCCs. All papillary RCCs showed TGR5 positive staining. No significant differences in TGR5 expression between type 1 and type 2 papillary RCCs were detected. Similarly, TGR5 staining may not be useful to differentiate chromophobe RCCs from oncocytomas.

## Conclusion

In summary, we employed immunohistochemistry to establish that TGR5 expression is robust in the distal convoluted tubules, thin loop of Henle, and collecting ducts. Among renal neoplasms, papillary RCCs, clear cell papillary RCCs, chromophobe RCCs and oncocytomas show various degrees of TGR5 expression. UCCs of the renal pelvis also strongly express TGR5. We demonstrate that the majority of clear cell RCCs, particularly cases without papillary features, are negative attributable to their curtailed expression of TGR5 mRNA. Overall, our data suggest that TGR5 staining may contribute to the diagnostic workup in distinguishing clear cell RCC from other renal tumors.

## Abbreviations

AMACR: Alpha methyl acyl coenzyme A racemase
CA-IX: Carbonic Anhydrase 9
RCC: Renal cell carcinoma
TGR5: G protein-coupled bile acid receptor
